# Expression of Concern: Investigations on segmentation-based fractal texture for texture classification in the presence of Gaussian noise

**DOI:** 10.1371/journal.pone.0352934

**Published:** 2026-07-02

**Authors:** The PLOS One Editors

After this article [1] was published, the following concerns were noted:

- The article uses terminology that differs from standards in the field (e.g. “squared blunder” instead of “squared error”, “neural system” instead of “neural network”, “haphazardly” instead of “randomly”).

In addition, the *PLOS One* Editors have concerns about the article’s compliance with the PLOS Authorship policy.

The corresponding author responded that the terminology issues arose due to language choices made during manuscript preparation that were not intended to misrepresent the research. However, the Editors remain concerned about the number and nature of these concerns.

In light of the cumulative issues, the *PLOS One* Editors issue this Expression of Concern. Readers are advised to interpret the article [1] with caution.
