# Supplementary material for: Investigations on segmentation-based fractal texture for texture classification in the presence of Gaussian noise
Source: PLoS One. 2025 Jan 10;20(1):e0315135. doi: 10.1371/journal.pone.0315135 (PMC11723552; doi:10.1371/journal.pone.0315135)
Supplement: Attachment — Submitted filename: Evaluation report-PONE-D-23-30849_reviewer.DOCX [file pone.0315135.s001.DOCX]

Report on

**Investigations on Segmentation-Based Fractal Texture for Texture Classification in the Presence of Gaussian Noise**

In this work, the authors in this paper assess two texture feature extraction methods based on SFTF and statistical moment-based texture features in the presence and absence of Gaussian noise. The SFTF and statistical moments-based handcrafted features are passed to a multilayer feed-forward neural network for classification. These models are evaluated on natural textures from Kylberg Texture Dataset 1.0. The results show the superiority of segmentation-based fractal analysis over other approaches. The average accuracy rates using the SFTF are 99% and 97% in the absence and presence of Gaussian noise, respectively. Although an interesting topic and an overall exciting work, the authors had to consider the following suggestions to improve the quality of their paper.

In the introduction part, it is important to mention previous research that involved texture classification but did not sufficiently address the problems caused by noise. As an example, cite the papers that concentrated on classical statistical moments or other learning techniques that work well on clean datasets but struggle with noisy inputs.

Regarding the contribution of the paper to be novel and more significant, the authors should mention why proposing this approach is significant. They should highlight the unique aspects of their approach compared to existing methods, for example. They mention 3 points as a contribution to their work, where just the first is considered as a contribution, and the other two points are the requirement to satisfy the first. It would be beneficial to clarify how these contributions are unique or how they advance this field.

All the symbols in the document should be written in an italic form. Please check section 2.2. The first paragraph in this section should be reformulated, it contains grammar mistakes.

In the second paragraph, the authors claim that “The higher value of the fractal dimension indicates a coarser texture.” Please justify this claim.

In Section 2.2, in the sentence “Due to this feature of SFTA”. What is SFTA?

The second paragraph should also be reformulated due to some mistakes, for example, 2^ed^.

The algorithm in the same page should be written in the standard form, many symbols should be defined, and other symbols should be written in one form. What is Th? What is [1……….|Th|-1]. Many words are written in a symbol form. Please, read the manuscript carefully and correct all the mistakes.

In Section 2.3, instead of rewriting the word that you need to define, replace it with “it”, for example:

“Smoothness: It calculates the relative smoothness of the gray intensities of a particular segment.

In Section 2.4, in my opinion, the first 3 paragraphs are fundamental text available in many documents. No need to mention them in these details.

The symbols in this section need to be adjusted. Also, step 10 needs more clarification.

Please enhance the figures with some detailed explanation and also the tables, please provide a detailed description of the structural details of the tables, especially Table 3, avoid the repetition of the Ref. and design the table in a more accurate form.

In the conclusion part, please highlight the novelty of your work, and how it improves the existing methods. What is the limitation of your work?

It would be interesting to explore and discuss practical applications beyond just theoretical discussion. This can make the research more impactful and relevant in these domains. It includes testing the proposed mappings in real-world scenarios.

The authors should read their paper many times to improve its quality and presentation.
