# Supplementary material for: Investigations on segmentation-based fractal texture for texture classification in the presence of Gaussian noise
Source: PLoS One. 2025 Jan 10;20(1):e0315135. doi: 10.1371/journal.pone.0315135 (PMC11723552; doi:10.1371/journal.pone.0315135)
Supplement: Attachment — Submitted filename: Response to Reviewers_revised.docx [file pone.0315135.s002.docx]

**Response to Reviewers**

**Reviewer #1**

**Comment:** Why is the word 'Investigation' associated with the title when only classification is performed as objective?
**Response:** It is used since multiple methods are used and the performance is investigated in presence of noise.

**Comment:** In material and methods, literature can be summarized, all mentioned equations and algorithms exist! Why is it re-written?
**Response:** We have summarized the literature and removed redundant equations and algorithms.

**Comment:** A generic MLP architecture was mentioned, instead model parameters should be presented.
**Response:** Model parameters have been presented instead of a generic MLP architecture in table 1.

**Comment:** Mentioned references achieved 99.78% accuracies:
**Response:** The work is based on deep learning based methods however our methodology is feature extraction based.

**Reviewer #2**

**Comment:** SFTF, although computationally complex, is also sensitive to noise. Clarity on addressing this issue may be included in result analysis.
**Response:** We have included a detailed analysis of the noise sensitivity of the SFTF algorithm in the results section.

**Comment:** Though the accuracy is less by 2% in the presence of Gaussian noise, what about the noise involved by the SFTF algorithm?
**Response:** We have discussed the impact of the noise introduced by the SFTF algorithm itself.

**Reviewer #3**

**Comment:** Mention previous research that involved texture classification but did not sufficiently address the problems caused by noise.
**Response:** We have included references to previous research that did not address noise problems sufficiently.

**Comment:** Clarify the unique aspects of your approach and how it advances the field.
**Response:** We have clarified the unique aspects of our approach and its contributions to the field.

**Comment:** All symbols should be written in italic form.
**Response:** All symbols have been converted to italic form.

**Comment:** Reformulate the first paragraph in section 2.2, and correct grammar mistakes.
**Response:** The first paragraph in section 2.2 has been reformulated and grammar mistakes corrected.

**Comment:** Justify the claim 'The higher value of the fractal dimension indicates a coarser texture.'
**Response:** The claim about fractal dimension and coarser texture has been justified.

**Comment:** What is SFTA? Define it in section 2.2.
**Response:** SFTA is corrected.

**Comment:** Reformulate the second paragraph in section 2.2, correct mistakes, and define symbols.
**Response:** The second paragraph in section 2.2 has been reformulated and symbols defined.

**Comment:** Replace repeated words with 'it' where appropriate in section 2.3.
**Response:** Repeated words have been replaced with 'it' where appropriate in section 2.3.

**Comment:** Remove fundamental text available in many documents in section 2.4.
**Response:** Fundamental text in section 2.4 has been removed.

**Comment:** Adjust symbols and clarify step 10 in section 2.4.
**Response:** Symbols in section 2.4 have been adjusted and step 10 clarified.

**Comment:** Enhance figures and tables with detailed explanations and avoid repetition.
**Response:** Figures and tables have been enhanced with detailed explanations and repetition avoided.

**Comment:** Highlight the novelty and limitations of your work in the conclusion.
**Response:** The novelty and limitations of our work have been highlighted in the conclusion.

**Comment:** Explore practical applications and discuss real-world scenarios.
**Response:** We have discussed practical applications and real-world scenarios.

**Comment:** Read the paper multiple times to improve quality and presentation.
**Response:** We have read and revised the paper multiple times to improve its quality and presentation.
