# Supplementary material for: Investigations on segmentation-based fractal texture for texture classification in the presence of Gaussian noise
Source: PLoS One. 2025 Jan 10;20(1):e0315135. doi: 10.1371/journal.pone.0315135 (PMC11723552; doi:10.1371/journal.pone.0315135)
Supplement: Attachment — Submitted filename: Minor revision 1 octt Response to Reviewers.docx [file pone.0315135.s003.docx]

**Response to Reviewers**

**Reviewer #1** Thanks for accepting the changes.

**Reviewer #2** Thanks for accepting the changes.

**Reviewer #3**: I see that the authors did not make all the required changes to improve their paper. The authors only answered that they had done what was required, but in fact, most of the changes were not made within the text. For example, if you look at Section 2.2, you will see that all the symbols are still in their incorrect forms and the ambiguous symbols have not been defined Equation 5 should be Equation (5) and all the equations must be written in standard form. Also, in the conclusions section, it is the same without any improvement. The researchers have highlighted Table 3. Why was it a shadow, and nothing has changed in the table? Where is the change that the researchers claimed they added in the introduction section, and why was it not highlighted? I ask the researchers to highlight the changes they made to the manuscript, and ask them to adhere to the required changes.

**Response:** Thanks for suggesting the changes for the improvement of the manuscript. I have checked carefully all the equations and corrected wherever required. All the Equation numbers are corrected as suggested. The abstract and conclusion are revised as suggested.
